# Low‐Level Cumulative Lead and Resistant Hypertension: A Prospective Study of Men Participating in the Veterans Affairs Normative Aging Study

**DOI:** 10.1161/JAHA.118.010014

**Published:** 2018-10-24

**Authors:** Alexander R. Zheutlin, Howard Hu, Marc G. Weisskopf, David Sparrow, Pantel S. Vokonas, Sung Kyun Park

**Affiliations:** ^1^ Department of Epidemiology University of Michigan Ann Arbor MI; ^2^ University of Michigan Medical School Ann Arbor MI; ^3^ School of Public Health, University of Washington Seattle WA; ^4^ Department of Epidemiology, Harvard T.H. Chan School of Public Health Boston MA; ^5^ VA Normative Aging Study Veterans Affairs Boston Health Care System Boston MA; ^6^ Department of Medicine Boston University School of Medicine Boston MA

**Keywords:** environment, epidemiology, hypertension, hypertension, high blood pressure, Cardiovascular Disease, Epidemiology, High Blood Pressure, Hypertension, Risk Factors

## Abstract

**Background:**

Bone lead offers a better method over blood lead measurement to discern long‐term lead exposure and accumulation. We examined the risk of resistant hypertension based on bone lead levels in a prospective cohort study of NAS (Normative Aging Study).

**Methods and Results:**

Participants had clinic data on hypertension (systolic blood pressure, diastolic blood pressure, and antihypertension medication), lead (blood, bone‐patella, bone‐tibia), and demographic and confounding variables. Cases of resistant hypertension were identified by meeting criteria for: (1) inadequate systolic blood pressure (>140 mm Hg) or diastolic blood pressure (>90 mm Hg) while taking 3 medications or (2) requiring >4 medications for blood pressure control. A modified Poisson regression was used for model analysis. Of the 475 participants, 97 cases of resistant hypertension (20.4%) were identified. Among the cases of resistant hypertension, the median tibia and patella lead levels were 20 μg/g and 25 μg/g, respectively, while median tibia and patella lead levels were 20 μg/g and 27.5 μg/g, respectively, in participants without resistant hypertension. Tibia lead demonstrated a significant association with resistant hypertension (relative risk, 1.19; 95% confidence interval, 1.01–1.41 [*P*=0.04]) per interquartile range increase in tibia lead (13–28.5 μg/g). Patella lead was not associated with resistant hypertension (relative risk, 1.10; 95% confidence interval, 0.92–1.31 [*P*=0.31]) per interquartile range increase in patella lead (18–40 μg/g). Blood lead levels were not significantly associated with resistant hypertension (relative risk, 1.11; 95% confidence interval, 0.88–1.40 [*P*=0.38]).

**Conclusions:**

Tibia lead represents a novel risk factor for resistant hypertension. Our study demonstrates an increased association between tibia lead and resistant hypertension status, with an increased risk of 19% per 1 interquartile range increase in tibia lead.

**Clinical Perspective**

**What Is New?**

- Cardiovascular sequelae may be contributed to by low levels of lead that do not have overt clinical manifestations.
- Tibia lead levels, available via a noninvasive well‐contained K shell X‐ray fluorescence measurement, may offer information about risk of development of resistant hypertension.
- Low‐level lead exposure, measured in the tibia, is associated with risk of development of resistant hypertension in a cohort of patients diagnosed with hypertension.

**What Are the Clinical Implications?**

- New knowledge on the health implications of low‐level exposure can help motivate exposure removal via infrastructure investment from a public health standpoint, as well as further factor in the exposome in predictive modeling from a clinical standpoint.
- Tibia lead is a novel biomarker for the risk of resistant hypertension and may offer greater insight into how low‐level lead impedes pharmacologic management of hypertension, as well as target more appropriate threshold for intervention.

## Introduction

Diagnosed hypertension continues to increase nationally, with ≈1 in 3 American adults affected.^1^, ^2^ The burden of hypertension costs society significantly. In 2014, hypertension was the leading cause of >410 000 deaths, with an estimated cost of $48.6 billion.^2^ Furthermore, the prevalence of hypertension is expected to be >40% of the US population by 2030.^3^ Given the magnitude of impact hypertension has on society, scientists and clinicians are using a multipronged approach to examine environmental, behavioral, and genetic risk factors to screen at‐risk populations. Managing risk occurs in concert with optimizing medical management, with the most recent recommendations published by the American College of Cardiology/American Heart Association Task Force.^4^

Despite rigorous clinical guidelines and risk mitigation, nearly half of people with hypertension lack appropriate control of their disease.^1^ This can be attributed to insufficient medical therapy, medication nonadherence and, lifestyle behaviors. However, resistant hypertension, as defined by persistent blood pressure (BP) above target while taking 3 antihypertension medications or controlled by >3 medications, remains a clinical burden.^5^, ^6^ Epidemiologic studies estimate that up to 20% of patients with hypertension will meet criteria for resistant hypertension, although rigorous review demonstrates a prevalence closer to 9% to 12%.^6^, ^7^ Behavioral factors, polypharmacy, pathophysiologic volume status, and secondary causes all contribute to the context that makes an individual resistant to proper pharmacotherapy for hypertension and subsequently at increased risk for cardiovascular mortality.^8^, ^9^

Lead has been studied for its potential role in elevated and volatile BP. Prolonged exposure to lead has been found to portend the development of hypertension.^10^ Cohort studies have linked blood lead levels to increased BP and hypertensive risk in England, Sweden, and the United States in both men and women.^11^, ^12^, ^13^, ^14^ Recently, blood lead has been shown to be a possible attributable risk in >400 000 cardiovascular‐related deaths annually.^15^ While strong epidemiologic evidence has been present for decades linking lead to elevation in BP, recent basic science research has provided insight into the mechanism of lead‐induced hypertension. In vitro and in vivo work points to lead interfering with systemic and local BP control via activity of the renin‐angiotensin system, sympathetic system, and vasocontractility properties of endothelium.^16^

The relationship between lead levels and BP, as well as mechanisms by which lead exerts influence on BP regulation, has been demonstrated in the literature. However, little work exists investigating the clinical burden of lead as it relates to hypertension. To our knowledge, no prior studies have assessed the relationship between cumulative lead exposure and risk of resistant hypertension. Given the interference lead can have on vascular regulation, atherosclerotic progression, and BP we hypothesize that the lead level in bone as a marker of cumulative lead exposure is an independent variable influencing the development of resistant hypertension.

## Methods

### Data Availability

To protect patient privacy, individual data cannot be made publicly available. Data are available upon request to qualified researchers from the Veterans Administration.

### Study Participants

All participants in the Veterans Affairs NAS (Normative Aging Study) are men and predominantly white. NAS is a longitudinal cohort study of 2280 male volunteers aged 21 to 80 years based out of the Boston Veterans Affairs Healthcare system initiated in 1963.^17^ In NAS, participants were seen in the clinic every 3 to 5 years for a complete history and physical, including measurement of BP and tracking of antihypertensive medical management. BP was measured using a standard mercury sphygmomanometer with an appropriately sized cuff. Patients were seated and then had systolic BP (SBP) and fifth‐phase diastolic BP (DBP) measured bilaterally to the nearest 2 mm Hg. Final recorded SBP/DBP values were the result of the average BPs from the right and left arm measurements. Beginning in 1991, blood and bone lead measurements were collected among participants who agreed to have those levels recorded. Given the current study focus on the development of resistant hypertension in patients with hypertension, participants were initially chosen if they were taking any medication for BP control or had elevated BP (SBP/DBP ≥140/90 mm Hg). Further inclusion criteria were based on the availability of data on hypertension (SBP, DBP, and antihypertension medication), lead (bone‐patella, bone‐tibia, blood), and demographic and confounding variables (listed below). Demographic and socioeconomic factors including age, race, education attainment, and income level, well‐documented confounders in the association between lead exposure and BP, were all obtained through NAS questionnaires.^10^, ^11^, ^12^, ^15^ Additionally, confounding variables including body mass index, cigarette smoking (pack‐years), and family history of hypertension were also obtained through NAS.

At the time of this study, 871 participants were identified to have information for hypertension and lead. Of these, 521 participants (59.8%) had a known diagnosis of hypertension, were receiving an antihypertensive agent, or had elevated SBP/DBP at the time of bone lead assessment. Forty‐two participants were additionally excluded because of incomplete information on covariates, yielding 475 as our final sample. Resistant hypertension status was determined without knowledge of bone lead levels. An individual was considered positive for resistant hypertension if they met the above criteria starting from one clinic visit before the date of initial bone lead measurement through the last year of data collected (from the period of 1986–2013). Individuals who were consented for NAS reported to the Ambulatory Clinical Research Center of the Brigham and Women's Hospital in Boston. All participants provided written informed consent. This study was reviewed and approved by the institutional review boards of each participating institute, the University of Michigan School of Public Health, the Harvard School of Public Health, and the Department of Veterans Affairs Boston Healthcare System.

### Tibia and Patellar Bone Lead Levels

Lead levels were measured at the mid‐tibia shaft and patella with K x‐ray fluorescence in NAS. Extensive review has validated K shell X‐ray fluorescence (KXRF) as a tool to determine bone lead levels.^18^, ^19^, ^20^ KXRF is a noninvasive method used to evaluate lead in bone via measurement of x‐ray traits correlating with fluorescent atoms of targeted elements. Measurements occurred via 30‐minute measurements of the mid‐tibia and patella. These data were correlated with each participant and assessed as separate variables (patella and tibia) to elucidate the correlative power of cortical (tibia) versus trabecular (patella) bone. The KXRF instrument also provided uncertainty measures, indications of precision of the bone lead measurements. Uncertainty measurements are calculated based on a goodness‐of‐fit calculation of various spectrum curves that are equivalent to a single SD.^10^, ^18^, ^20^ To ensure the quality of the KXFR measurements, tibia and patella bone measurements with uncertainty >10 μg and 15 μg per gram of bone mineral, respectively, were excluded from our analysis.

### Blood Lead Levels

Blood samples were analyzed via graphite furnace atomic absorption with Zeeman background correction (ESA Laboratories, Inc). Values <1 μg/dL were coded as 0 (<1%). Following every 20 samples, the instrument was calibrated with National Institute of Standards and Technology Standard Reference Material (955a, lead in blood). Reference samples from the Centers for Disease Control and Prevention (Atlanta, GA), precision, measured as coefficient of variation ranged from 8% (when blood lead levels were 10 to 30 μg/dL) to 1% (when blood lead levels were >30 μg/dL).

### Resistant Hypertension

During each Veterans Affairs visit, full clinical data were collected, including information about individuals’ BP (measurement methodology noted above) and medication list. For the current study, resistant hypertension was defined as inadequate BP control while taking ≥3 antihypertensive medications of different classes (diuretic, β‐blocker, calcium channel blocker, α‐inhibitor, angiotensin‐converting enzyme inhibitor, angiotensin receptor blocker, or mineralocorticoid antagonist) or adequate control while taking ≥4 antihypertensive medications. The cutoff for BP control was set at 140 mm Hg SBP and 90 mm Hg DBP.^6^, ^8^ Given the time of this study, SBP and DBP thresholds of 140 mm Hg and 90 mm Hg with medication quantity, as opposed to exact regimen composition, were used as cutoff criteria for resistant hypertension.

### Statistical Analysis

Initial analysis examined descriptive statistics. We determined medians (interquartile ranges [IQRs]) for lead measures, as well as for other continuous variables, and percentages for categorical variables based on the cohort and on resistant hypertension status. Pearson correlation was calculated for the relationship between tibia lead and patella lead, as well as blood lead with tibia and patella lead, respectively. A modified Poisson regression with a robust error variance, the efficient approach recommended to estimate relative risk (RR) in prospective studies with a binary outcome, was used to assess the relationship between resistant hypertension status and tibia and patella bone lead levels, as well as blood lead leavels.^21^ Significance was defined as a *P* value ≤0.05 for all analyses. We computed the RRs for an IQR increase (ie, from the 25th to the 75th percentile) for each lead marker as exp(IQR×β), and with 95% confidence intervals (CIs) as exp(IQR×(β±1.96×SE)), where β and SE are the estimated regression coefficient and its standard error. Analysis was adjusted for demographic and confounding variables including age, race/ethnicity, education attainment, income level, body mass index, family history of hypertension, and cigarette smoking (pack‐years). Race/ethnicity was collapsed into white and nonwhite, given the few black and Hispanic participants, and subsequently referred to as race. Additionally, income was categorized into 4 groups based on quartile of annual income earned: <$6000, $6000 to $8599, $8600 to $9999, and >$10 000. Given the nature of income data, the cutoffs were not exactly in each quartile and therefore actual frequencies in each group were not equal to 25%. Sensitivity analysis was conducted for smoking status in combination with pack‐years. Smoking status was defined as never, current, or former (having quit before baseline of the current study). Statistical analyses were performed using the geepack package in R 3.4.3 GUI 1.70 El Capitan build (7463).

## Results

**Figure 1:**
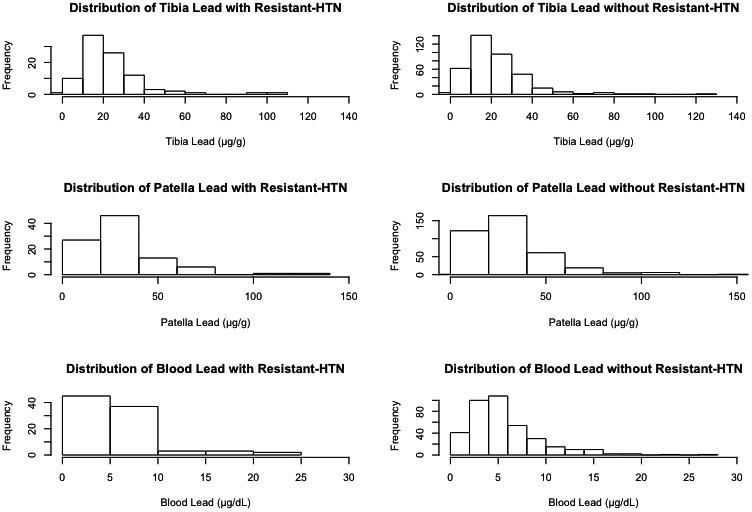
Histogram of lead by source. Distribution of tibia, patella, and blood lead levels by resistant hypertension (HTN) status, as seen left to right.

Participants were predominantly white (96.8%) and ranged in age from 48 to 93 years (mean age, 68.2 years) as seen in Table 1. Median lead levels were 20 μg/g (IQR, 13–28.5 μg/g) for the tibia and 27 μg/g (IQR, 18–40 μg/g) for the patella. Distributions of tibia, patella, and blood lead levels are shown in Figure 1. Tibia and patella lead were strongly correlated (*r*=0.78; 95% CI, 0.75–0.82 [*P*<0.001]). Median blood lead levels were 5.0 μg/dL (IQR, 3.36–8.00 μg/dL) and were moderately associated with tibia lead levels (*r*=0.38; 95% CI, 0.30–0.46 [*P*<0.001]) and patella lead levels (*r*=0.43; 95% CI, 0.35–0.50 [*P*<0.001]). Age, smoking (pack‐years), education, and race were significantly associated with high tibia lead levels (*P*<0.05), and age, smoking (pack‐years), education, and race were significantly associated with higher patella lead levels (*P*<0.05).

**Table 1:** Descriptive Statistics by Total Sample and Resistant Hypertension Status

	All	Resistant Hypertension	
		Yes	No
No. (%)	475 (100)	97 (20.4)	378 (79.6)
Tibia lead, median (IQR), μg/g	20.0 (13.0–28.5)	20.0 (15.0–29.0)	20.0 (13.0–28.0)
Patella lead, median (IQR), μg/g	27.0 (18.0–40.0)	25.0 (19.0–39.0)	27.5 (18.0–40.0)
Blood lead, median (IQR), μg/dL	5.0 (3.4–8.0)	5.0 (3.0–8.0)	5.00 (4.0–7.8)
Age, median (IQR), y	67.9 (63.2–72.6)	66.6 (62.3–70.1)	68.1 (63.5–73.0)
BMI, median (IQR), kg/m^2^	27.8 (25.6–30.4)	28.7 (26.6–31.2)	27.6 (25.4–30.3)
Pack‐years of smoking, median (IQR)	13.6 (0.0–34.0)	8.8 (0.0–25.5)	15.7 (0.0–36.0)
Smoking status, %			
Never	28.8	30.9	28.3
Current	4.8	4.1	5.0
Former	66.3	64.9	66.7
Annual income, %			
<$6000	29.3	29.8	29.4
$6000–$8599	30.5	31.9	30.2
$8600–$9999	18.1	19.1	17.8
≥$10 000	22.1	19.1	22.8
Race, white, %	96.8	96.9	96.8
Education, %			
Grade school	0.2	0.0	0.3
High school dropout	8.6	8.5	8.7
High school graduate	36.2	34.0	36.7
Technical support	10.9	8.5	11.5
College dropout	13.5	23.4	11.0
College graduate	17.9	13.8	18.9
Graduate school	5.5	4.3	5.8
Professional school	7.2	7.4	7.1

BMI indicates body mass index (calculation of weight in kilograms divided by the square of height in meters); IQR, interquartile range.

Of the 475 total study participants with hypertension, 97 cases of resistant hypertension were identified with the associated bone lead measurements, equaling 19.8% of all study participants. Among the cases of resistant hypertension, median tibia lead was 20 μg/g and median patella lead was 25 μg/g. Study participants without resistant hypertension had a median tibia lead level of 20 μg/g and median patella lead level of 27.5 μg/g.

**Figure 2:**
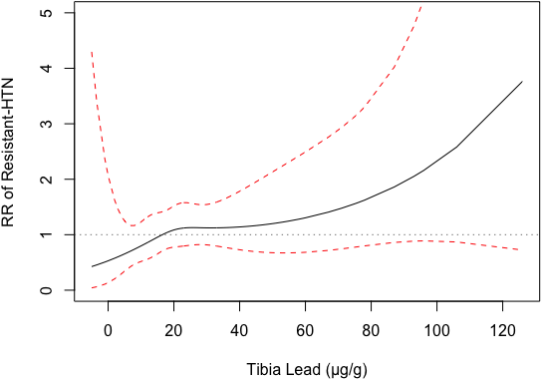
Dose‐response curve of tibia lead level and resistant hypertension (HTN) risk. Dose‐response plot demonstrating observed tibia lead levels when compared with change in relative risk of resistant hypertension outcome utilizing the final adjusted model. Red dashed lines indicate 95% confidence interval. Grey dotted line indicates relative risk of 1.

Final adjusted models accounting for family history of hypertension exhibited significance in the relationship between tibia and resistant hypertension (RR, 1.19; 95% CI, 1.01–1.41 [*P*=0.04]; model 3, Table 2), but not patella lead on resistant hypertension status (RR, 1.10; 95% CI, 0.92–1.31 [*P*=0.31]; model 3, Table 2). As seen in Figure 2, there is a linear dose‐response relationship with tibia lead. There is a steeper association observed in lower concentrations of tibia lead, notably from 0 to 20 μg/g (Figure 2). Sensitivity analysis for smoking status (current smoker, former smoker, or never smoker) added to the fully adjusted model did not significantly alter the relationship between tibia or patella lead with resistant hypertension status (RR, 1.19; 95% CI, 1.01, 1.41 [*P*=0.04] and RR, 1.09; 95% CI, 0.92–1.30 [*P*=0.31]; data not shown). Results for sequential models (model 1: age, body mass index, smoking [pack‐years]; model 2: model 1 plus education, income, and race) are shown below in Table 2.

**Table 2:** Association Between Bone and Blood Lead and Resistant Hypertension Among Participants in the Normative Aging Study

	β‐Coefficient	SE	RR (95% CI)	*P* Value
Tibia models				
Model 1	0.009	0.005	1.13 (0.98–1.32)	0.099
Model 2	0.011	0.005	1.18 (0.99–1.39)	0.053
Model 3	0.012	0.005	1.19 (1.01–1.41)	0.038
Patella models				
Model 1	0.003	0.003	1.07 (0.91–1.27)	0.412
Model 2	0.004	0.004	1.09 (0.92–1.29)	0.322
Model 3	0.004	0.004	1.10 (0.92–1.31)	0.312
Blood models				
Model 1	0.015	0.024	1.08 (0.86–1.36)	0.584
Model 2	0.019	0.005	1.10 (0.88–1.39)	0.397
Model 3	0.019	0.024	1.11 (0.88–1.40)	0.349

β‐Coefficient refers to log‐transformed effect estimate of resistant hypertension based on a single unit increase in exposure (tibia lead, patella lead, or blood lead). Relative risk (RR) represents the ratio of cumulative incidence for a 1 interquartile range increase in exposure (15.5 μg/g for tibia lead, 22.0 μg/g for patella lead, or 4.6 μg/L for blood lead). *P* value represents the significance of the association between exposure (tibia lead, patella lead, or blood lead) and resistant hypertension for a RR >1.00. Model 1 was adjusted for body mass index, age, and smoking (pack‐years). Model 2 includes model 1 plus annual income by quartile, educational attainment, and race. Model 3 includes model 2 plus family history of hypertension. CI indicates confidence interval; SE, standard error.

Whereas tibia bone lead demonstrated a significant association with resistant hypertension, this was not found when examining baseline blood lead levels as the exposure. Final covariate adjustment for blood lead was not found to be significantly associated with resistant hypertension (RR, 1.11; 95% CI, 0.88–1.40 [*P*=0.38]) (Table 2).

## Discussion

For decades, lead has been studied for the nefarious effect it can have on multiple organ systems. While the clinical implication of lead exposure on neurological development has been well studied, less is known about how lead impacts clinical outcomes from a cardiovascular standpoint. The statistically significant relationship between bone lead and BP is well established; however, there is a paucity of research supporting a clinically relevant relationship. Our study demonstrates the association of cumulative lead burden, as measured by cortical bone in the tibia, with development of resistant hypertension. After accounting for body mass index, age, race, income, education, cigarette smoking (pack‐years), and family history, we found a significant association between tibia bone lead levels, although not patella or blood lead, and resistant hypertension among men participating in NAS. To our knowledge, this is the first study that posits cumulative lead exposure as a risk factor for resistant hypertension development.

While blood lead measurements offer clinicians insight into relatively recent exposure and toxicity, blood lead levels measured in circulation represents only 5% to 15% of total body lead.^18^ Ingested lead distributes extensively throughout the body and can be found deposited in various tissue types and organs. Lead's similarity to other cations, such as calcium, allow it to compete in similar biochemical pathways.^22^ Bone lead levels account for ≈85% to 95% of total body amounts.^18^ Additionally, the half‐life of lead in cortical bone is 25 to 30 years, and only 1 month in blood.^19^ Bone serves as a cache for lead and accounts for 40% to 70% of measured levels in blood predominantly as a result of turnover in trabecular regions.^23^

Bone serves as a particularly expansive reservoir for lead because of the high calcium requirement in the process of hydroxyapatite formation during bone mineralization. Hydroxyapatite composes a majority of bone mass, and lead can integrate into position normally filled by calcium. While trabecular bone reveals higher enrichment of lead and more rapid release into circulation, lead levels in cortical bone better represent cumulative lead dose due to the prolonged elimination half‐life.^24^ Individuals faced with greater exposure and hydroxyapatite capture of lead during the mineralization process are vulnerable to greater absolute lead deposition in cortical bone, as well as circulatory release from trabecular bone.^18^, ^24^

Lead has been shown to interfere with the function of vasculature and endothelial cells through increases in renin‐angiotensin system and vasoconstricting prostanoids, in combination with decreases in the potent vasodilator, nitric oxide.^25^ In vivo research has focused on the mechanism by which lead influences BP. Low levels of lead leads to a marked decrease in nitric oxide bioavailability secondary to anion superoxide production. Furthermore, lead upregulates renin‐angiotensin system activity via increased angiotensin‐converting enzyme and angiotensin II receptor type 1, as well as greater production of cyclooxygenase‐derived prostanoids, which results in arterial contraction.^25^ Vasoconstriction results in a further rise in BP.

In addition to effects on local vasodilatory properties and renin‐angiotensin system activity, lead levels can also cause desensitization of ß‐adrenergic receptors, which are widely distributed throughout the body. ß_2_‐receptors serve as arterial targets, which result in vasodilation and a decrease in systemic BP upon activation.^26^ In rat models of lead‐exposure and lead‐removal studies, in vivo analysis revealed a significant reduction in ß‐adrenergic receptor as the lead load became larger.^26^, ^27^ Understanding the relationship by which cumulative lead deposition impedes control of hypertension can aid in assessing patient risk.^28^ Furthermore, hypertensive risk can be reduced by effective lifestyle.^29^ The addition of mitigating environmental risk factors, such as lead, that increase clinical risk of resistant hypertension may prove to be another piece of the puzzle in lowering the burden of hypertensive disease burden and complications.

Public health efforts to reduce lead exposure have been successful in lowering lead levels among all age demographics.^30^, ^31^ However, clinical management remains largely targeted toward acute toxicity.^31^ The threshold for acceptable blood lead levels is based off of historical population distributions, yet no known normal level exists.^32^ With higher fidelity measurement devices, we can now determine the subtle way in which long‐term exposure to lead relates to poor health outcomes. This knowledge has prompted calls for lowering the threshold of acceptable lead levels and the level of intervention.^32^, ^33^, ^34^ Vigilant screening and exposure reduction remains the primary method to reduce lead stores.^35^, ^36^ Certain therapeutic interventions, such as calcium supplementation, may offer additional actionable items in reducing lead levels and pathologic sequelae.^37^ The current study and other work demonstrating the relationship between lead and hypertension suggests that investigation into more aggressive screening, exposure reduction, and intervention at the individual level may prove beneficial in certain patients with high cardiovascular risk.^10^, ^11^, ^12^, ^13^, ^18^, ^37^, ^38^

### Study Limitations

There are key limitations to note in the current study. Patients categorized as meeting criteria for resistant hypertension may experience uncontrolled hypertension attributable to secondary causes uncaptured by the current study. Because of the method by which resistant hypertension was defined and the variability of office BP measurements, we can only ascertain that cases are “apparent resistant hypertension.” This leaves the possibility of certain identified resistant hypertension cases actually being captured secondary causes of treatment resistance or isolated elevations in BP that could otherwise be captured by ambulatory BP monitoring.^39^ However, our study sample is similar to the estimate of 10% to 20% of those diagnosed with hypertension meeting criteria of resistant hypertension and does not appear to exceed population expectations. Future studies utilizing ambulatory BP monitoring and incorporating diagnosis that can cause apparent resistant hypertension would allow for more accurate classification of true resistant hypertension.

Among our 3 exposure variables (tibia, patella, and blood leads), only tibia demonstrated a significant association. Lead levels in both the tibia and patella have previously been demonstrated to be positively associated with hypertension risk and cardiovascular outcomes.^10^, ^20^ KXRF precision has been validated for bone lead levels >10 μg in clinical settings, and even <10 μg in research settings.^18^ However, there is greater measurement precision for tibia bone lead due to bone mineral density, in comparison to patella bone lead.^18^ The relative difference in precision of tibia versus patella lead levels via KXRF may also influence the reliability of tibia as a viable biomarker. Our model did not demonstrate a statistically significant association between patella lead and resistant hypertension, only tibia lead and resistant hypertension. However, both sources of lead had a positive association with risk of resistant hypertension, which is consistent with prior literature.^10^, ^20^

All participants in NAS are men, and primarily white, which reduces the generalizability of this study. A more representative cohort is necessary to assess the relationship between bone lead and resistant hypertension across various demographics. Additionally, we included a relatively small sample size, 475 participants, which may decrease our power to detect effect size and increases the risk of type I error. The final cohort may be subject to selection bias, as there is higher mortality associated with resistant hypertension. The inherent mortality faced by participants with the outcome of interest may cause loss of follow‐up before full data collection. This limitation reduces the certainty with which we can draw conclusions from the current study.

## Conclusions

Lead has no known physiologic role in the body and historical lead levels for clinical intervention have been based on population standard distributions and presentation of acute toxicity. However, the cumulative effect of low‐level lead may manifest through more subtle mechanisms of slow interference with biochemical systems involved in vascular contractility and volume management. This is the first study, to our knowledge, that points to cumulative low‐level lead exposure as a potential risk factor for resistant hypertension. Further reduction in lead exposure and burden may improve BP management and reduce the prevalence of resistant hypertension.

## Sources of Funding

This work was supported by grants from the National Institute of Environmental Health Sciences R01‐ES005257, K01‐ES016587, and P30‐ES017885, and by the Centers for Disease Control and Prevention/National Institute for Occupational Safety and Health grant T42‐OH008455. The Veterans Affairs Normative Aging Study is supported by the Cooperative Studies Program/Epidemiology Research and Information Center of the US Department of Veterans Affairs and is a component of the Massachusetts Veterans Epidemiology Research and Information Center, Boston, Massachusetts.

## Disclosures

None.
